# Household Air Pollution Exposure and Influence of Lifestyle on Respiratory Health and Lung Function in Belizean Adults and Children: A Field Study

**DOI:** 10.3390/ijerph13070643

**Published:** 2016-06-28

**Authors:** Stephanie P. Kurti, Allison N. Kurti, Sam R. Emerson, Richard R. Rosenkranz, Joshua R. Smith, Craig A. Harms, Sara K. Rosenkranz

**Affiliations:** 1 Department of Kinesiology, Kansas State University, 1A Natatorium, Manhattan, KS 66506, USA; smith424@ksu.edu (J.R.S.); caharms@ksu.edu (C.A.H.); 2 Department of Psychiatry, University of Vermont, Burlington, VT 05401, USA; akurti@uvm.edu; 3 Department of Food, Nutrition, Dietetics and Health, Kansas State University, Manhattan, KS 66506, USA; same@ksu.edu (S.R.E.); ricardo@ksu.edu (R.R.R.); sararose@ksu.edu (S.K.R.)

**Keywords:** pulmonary function, lifestyle, physical activity, indoor air pollution, household air, respiratory physiology, Belize, field study

## Abstract

Household air pollution (HAP) contributes to the global burden of disease. Our primary purpose was to determine whether HAP exposure was associated with reduced lung function and respiratory and non-respiratory symptoms in Belizean adults and children. Our secondary purpose was to investigate whether lifestyle (physical activity (PA) and fruit and vegetable consumption (FV)) is associated with reported symptoms. Belizean adults (*n* = 67, 19 Male) and children (*n* = 23, 6 Male) from San Ignacio Belize and surrounding areas participated in this cross-sectional study. Data collection took place at free walk-in clinics. Investigators performed initial screenings and administered questionnaires on (1) sources of HAP exposure; (2) reported respiratory and non-respiratory symptoms and (3) validated lifestyle questionnaires. Participants then performed pulmonary function tests (PFTs) and exhaled breath carbon monoxide (CO). There were no significant associations between HAP exposure and pulmonary function in adults. Increased exhaled CO was associated with a significantly lower forced expiratory volume in 1-s divided by forced vital capacity (FEV_1_/FVC) in children. Exposed adults experienced headaches, burning eyes, wheezing and phlegm production more frequently than unexposed adults. Adults who met PA guidelines were less likely to experience tightness and pressure in the chest compared to those not meeting guidelines. In conclusion, adults exposed to HAP experienced greater respiratory and non-respiratory symptoms, which may be attenuated by lifestyle modifications.

## 1. Introduction

Household air pollution (HAP) refers to the various toxins (e.g., carbon monoxide (CO), sulfur dioxides) that come from household sources (e.g., stoves, poorly ventilated homes, mosquito coils). The most common source of HAP in developing countries is the burning of biomass fuels [1], and many individuals living in underdeveloped countries burn these fuels inside homes via open fires or stoves that are often poorly functioning [2,3]. Although most HAP research has focused on the toxins emitted from open fires, there are numerous other sources of HAP, including the incomplete combustion of fuels or cheap fuel used in electric stoves, poorly ventilated homes without chimneys, active and/or passive exposure to tobacco smoke [2,3], and mosquito coils (pyrethrum coils emitting formaldehyde) commonly burned during summer nights to prevent insect bites [4].

Exposure to one or more of the above sources may contribute to approximately 3% of the global burden of disease [5]. Strong evidence suggests a relationship between exposure to these sources and lower respiratory tract infections in children under five [6], chronic obstructive pulmonary disease (COPD) [7], lung cancer in women over 30 years of age [8], and associations between exposure to these sources and both asthma and tuberculosis [8]. Several other problems that can result from exposure to these sources are increased airway responsiveness [9], airway inflammation [10], and increased prevalence of adverse respiratory symptoms (e.g., burning eyes, wheeze, difficult breathing, coughing). Particulate matter (PM) emitted from household sources has detrimental consequences to lung health; although CO in the exhaled breath has been used as an acceptable proxy for exposure to household sources [11].

Aside from studying a single source of CO exposure and associated respiratory symptoms, the use of spirometry (the gold standard for evaluating lung function [12,13]) is sparse in existing literature. Additionally, the majority of existing literature has focused on respiratory symptoms as the primary consequence of exposure, though research has shown that CO exposure is also related to various non-respiratory symptoms, such as eye discomfort, headache and back pain [14]. Exposure is also commonly indexed using self-reported measures [15] rather than quantified objectively (e.g., measuring breath CO output). Thus, the main purpose of our study was to evaluate the relationship between HAP exposure, reported respiratory and non-respiratory symptoms, and lung function.

In addition, no existing research, to our knowledge, has assessed lifestyle factors (e.g., diet and physical activity (PA)) in people in Belize to determine if they are associated with respiratory and non-respiratory symptoms based on HAP exposure. Therefore, we also sought to determine if the participants’ lifestyle might modify their symptoms when exposed to HAP. Fruits and vegetables have antioxidant properties that may combat the oxidative stress and therefore protect against subsequent inflammation [16] from HAP exposure. Inadequate fruit and vegetable (FV) consumption is also correlated with decreased lung function in children [17]. Increasing FV consumption [18] as well as dietary antioxidants [19] have been shown to improve post-exercise lung function. Previous research has reported PA is a natural antioxidant, and individuals who meet and exceed PA guidelines (≥150 moderate-vigorous PA/week) have an increased capacity to neutralize free radicals [20]. Additionally, PA may reduce airway responsiveness [21] and asthma risk [22]. Therefore, a secondary purpose of this study was to investigate whether PA and FV consumption was associated with reported respiratory and non-respiratory symptoms in Belizeans. We hypothesized that individuals exposed to HAP sources would have the highest exhaled CO and respiratory and non-respiratory symptoms, and the poorest lung function. Further, we hypothesized that individuals who did not meet FV consumption or PA recommendations would have the highest reported respiratory and non-respiratory symptoms.

## 2. Materials and Methods

### 2.1. Participants

Belizean adults (*n* = 67, 19 Male (M)/48 Female (F)) and children (*n* = 23, 6 M/17 F) participated in the study. Verbal consent was obtained from all participants. The study was approved by the Belizean Department of Health and the Institutional Review Board Involving Human Subjects at Kansas State University (#6741); the study conformed to the Declaration of Helsinki principles. The study was conducted in San Ignacio, Belize, and surrounding areas. The study area comprised eight rural villages of low and middle-income families. All data collection was conducted at free, walk-in clinics organized by ProWorld Service (www.proworldsc.org, San Ignacio, Belize). Clinics were run by ProWorld Service volunteers, and a medical doctor was on site at all times. Data collection took place over a two-week period during summer.

### 2.2. Study Design

**Figure 1:**
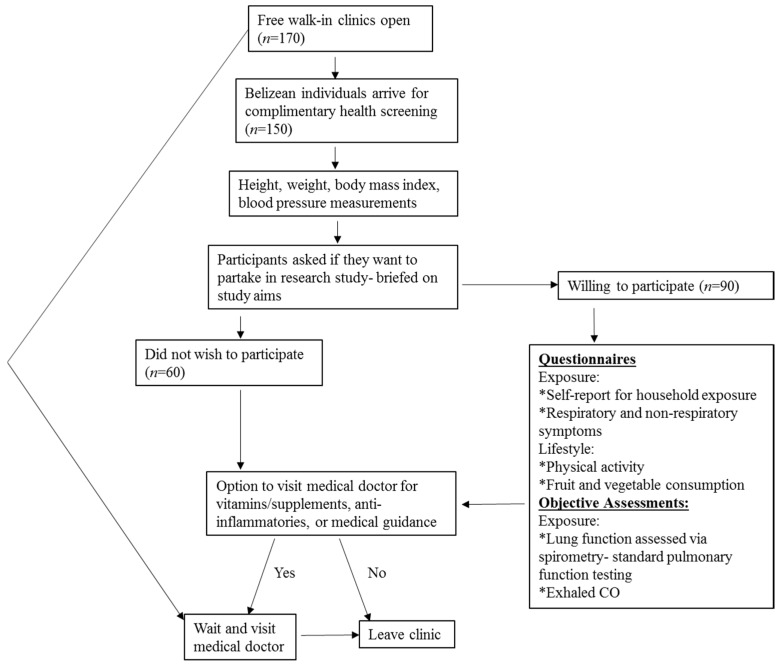
Schematic of the study design at the free walk-in clinics.

Free walk-in clinics were organized by ProWorld to offer complimentary health screenings to participants in Belize. Health screenings included height, weight, body mass index (BMI), and blood pressure measurements. The Belizeans willingly attended the clinics to receive the free health screening and/or meet with the supervising physician to receive vitamins/anti-inflammatories and/or supplements. Upon arrival to the walk-in clinics, ProWorld volunteers collected height and weight to calculate BMI, and then measured blood pressure. Next, clinic attendees were offered the opportunity to participate in a research study investigating HAP and respiratory health. If the clinic attendee did not wish to participate, they either received the health screening only or visited with the doctor on site to receive the vitamins/supplements, anti-inflammatories or receive additional medical guidance. Participants were required to receive the health screening prior to enrollment in the research study. Individuals were excluded from the research study if they only came to the clinic to visit the doctor. If the clinic attendee chose to participate, they completed the questionnaires and lung function measurements after the health screening. The schematic for the study design is displayed in Figure 1.

### 2.3. Exposure, Respiratory and Non-Respiratory Symptoms

Participants first completed the self-reported exposure questionnaire to determine the number and types of HAP sources that they and their children (if applicable) had been exposed to over the past year. All participants indicated they had lived in their current homes for at least one year at the time of the study. They were then asked to report frequency of reported respiratory and non-respiratory symptoms [14,15]. Questions on airway symptoms (i.e., cough, tightness in chest, wheeze, and phlegm) were developed from standard questions on COPD and asthma used in previous HAP research [23]. Non-respiratory symptoms (e.g., headache) were items previously reported on questionnaires with HAP exposure [14]. The surveys listed the respiratory and non-respiratory symptoms on a Likert scale (0 = never experience; 1 = occasionally (have experienced before); 2 = sometimes (1–2 times per month); 3 = frequently (1–2 times per week) 4 = often (3–5 times per week); 5 = very often (experience daily).

### 2.4. Lifestyle Assessment

The Physician-based Assessment and Counseling for Exercise + Physical Activity (PACE + PA) required participants to identify how many days of both the past week and a typical week in which they were active for at least 60 min per day for children (≥5 checked boxes) [24] and ≥3 checked boxes satisfied the physical activity requirement for adults. The Physician-based Assessment and Counseling for Exercise + Fruit and Vegetable consumption (PACE + FV) required participants to identify how many servings of fruit they consumed per day, as well as how many servings of vegetables they consumed per day [25]. An additive score of less than 5 indicated they did not meet the five-a-day guideline for FV consumption of five servings of fruits and vegetables per day [25].

### 2.5. Pulmonary Function Tests

Pulmonary function tests (PFTs) were performed on a portable spirometer (MIR Winspiro Pro, Waukesha, WI, USA) to measure lung function. The maximum flow volume loop was used to determine expiratory flow (PEF), forced expiratory flow in 1-s (FEV_1_), forced vital capacity (FVC), and forced expiratory flow at 25%–75% of vital capacity (FEF_25%–75%_). PFTs were performed according to American Thoracic Society/European Respiratory Society (ATS/ERS) guidelines [12]. Participants performed maximal flow volume loops until they could consistently produce three measurements within 10% of one another, and the best measurement was used for analysis [12]. Individuals performed blows until three measurements of FEV_1_, FVC, FEF_25%–75%_, and PEF were consistent. As soon as three measurements were within 10%, the best was chosen for analysis as stated in the standardization of spirometry in Miller et al. (2005). To achieve an acceptable maximum flow volume loop (MFVL), (1) the expiratory volume in FEV_1_ must be <5% of the FVC or 0.150 L, whichever is greater; (2) there must be no cough during the first second of the expiratory portion; (3) the participant does not inhale too early (test does not terminate early); (4) there is no hesitation during the maneuver which may preclude an accurate measurement of FEV_1_ and FVC; (5) there is no leak or obstruction in the mouthpiece and (6) the maneuver is performed correctly without an extra breath taken. Eight consecutive blows was the upper limit, and no subjects exceeded eight consecutive blows. No adults or children showed a drop in FEV_1_ that exceeded 20% while performing the maneuver multiple times. Percent of predicted was calculated based off of reference values [13].

### 2.6. Exposure to Pollutants

Exposure was assessed both through objective measures and through a self-report instrument. Our objective measure of exposure to HAP was participant’s breath CO output, which was collected using a handheld breath CO monitor (Bedfont Scientific Ltd., Kent, UK). Exhaled CO has been used as a proxy of objectively measuring exposure to HAP [11,26,27]; however, it is indicative of the most recent source of exposure and therefore direct measures are preferred when they are possible. In this field study, direct measures of exposure were not possible in the Belizean clinics and that is why self-reported measures were also included. Still, exhaled CO levels are highly correlated with carboxyhemoglobin levels and therefore is an acceptable method of exposure assessment [26]. Considering assessment of exposure via exhaled CO poses limitations, self-reported measures of exposure were included, which were based on Diaz’s surveys [14,15]. Participants reported the number of sources that they were exposed to, and the frequency with which they experienced respiratory and non-respiratory symptoms. Participants with exhaled breath CO > 9 ppm were identified as exposed (defined by the 8-h average Environmental Protection Agency (EPA) standard [28]), as were participants who reported exposure to one or more of the CO sources that we assessed (i.e., mosquito coils, traditional wood-burning stoves, passive or active tobacco smoke). Therefore, in our analyses, it was possible for a participant to have an exhaled CO > 9 ppm and report as having no sources of exposure. As mentioned previously, the EPA has identified this level of CO as a cut point for exposure [28]; however, usually, studies include exhaled CO measurements in the home as well to assess multiple sources of exposure [11,27]. Considering this was not possible in our clinical work, we sought to determine whether there were relationships between household air pollution and resulting health outcomes based on both previously established EPA cut points for CO as well as self-report to exposure sources.

### 2.7. Data Analysis

Data analyses were performed using SPSS Software v22.0 (IBM Corporation, Armonk, NY, USA). Independent samples *t*-tests were conducted to determine whether adults’ PFT values differed as a function of exposure (based on self-report and EPA cut points). Data were not normally distributed for children, so Mann-Whitney U tests were conducted to determine whether PFT indices differed in exposed versus unexposed groups. To test associations between the proportion of people with respiratory and non-respiratory symptoms and exposure, Pearson correlations were performed. Chi-squared tests were performed and where cell counts were <5, Fisher’s exact tests were used. For all tests, the *p-*value was set to < 0.05 to determine significance. Data are presented as means ± standard deviation (SD). Preliminary analyses indicated that there were no differences in primary outcome variables between sexes or sites of clinics, and therefore results were not stratified based on these variables.

## 3. Results

### 3.1. Participants

Sixty-seven adults (19 M/48 F) and 23 children (6 M/17 F) were sampled at the following sites: Kontiki, Awe Village, Santiago, Backstreet, Santa Elena, San Ignacio, Cayo, and Santa Maria. Adults were 44.2 ± 16.1 years old, and had a BMI of 30.4 ± 6.1 kg/m^2^. Thirty-one adults (46.3%) met current PA requirements, and five (7.5%) met current fruit and vegetable consumption guidelines. Children (individuals < 18 years of age) were 11.7 ± 3.1 years old. Nine children (39.1%) met current PA requirements, and one child met current fruit and vegetable consumption guidelines. Demographic and health data for adults and children are displayed in Table 1.

### 3.2. Exposure According to EPA Cut Points

When exposure was defined based on the criteria set forth by the EPA (>9 ppm), 19 adults were classified as exposed (mean CO = 24.2 ± 24.4 ppm) and 12 were unexposed (mean CO = 4.1 ± 2.5 ppm) (*p* = 0.009). Using EPA exposure criteria, 10 children were unexposed (average CO = 4.4 ± 1.6 ppm) and 2 were exposed (average CO = 11.0 ± 1.4 ppm) (*p* < 0.0001).

### 3.3. Exposure According to Self-Report

Thirty-one adults (46.3%) reported that they were exposed to HAP. This included any HAP exposure source (mosquito coils, traditional wood-burning stoves) as well as tobacco smoke. There were a total of eight participants (of the 31 total) who were current smokers, had previously smoked tobacco products, or lived with a smoker (i.e., passive exposure). Among those adults who were exposed according to self-report, the mean CO was 19.7 ± 24.7. Self-reported unexposed adults (*n* = 10) had a mean CO of 9.9 ± 3.8. Therefore, using self-report, exposed adults did not have significantly greater exhaled CO (*p* = 0.084), but did when EPA cut points were used (*p* = 0.009). Eight children reported exposure to HAP with a mean CO of 5.9 ± 3.6 ppm. The four unexposed children had a mean CO of 4.8 ± 1.3 ppm, which was not significantly different between exposure groups (*p* = 0.448).

### 3.4. Lung Function Data

Lung function based on self-reported exposure is included in Table 2. Using either self-reported exposure or EPA-identified exposure >9 ppm of exhaled CO, there were no PFT differences in the percent of predicted lung function between exposed versus unexposed adults (CO > 9 ppb: FEV_1_; *p* = 0.551, FVC; *p* = 0.468, FEV_1_/FVC; *p* = 0.086. FEF_25%–75%_, *p* = 0.483, PEF; *p* = 0.399). Additionally, all adult predicted values were within normal ranges [12]. Thirteen of 23 children received lung function tests (5 = unexposed, 8 = exposed). One of the children who reported as exposed was not able to complete measurements of CO. In children, mean PFT values did not differ as a function of exposure. Lung function data based on self-report are included in Table 3. Given that only two children were exposed based on EPA criteria, and because there was little variability in children’s CO, comparisons were not made between EPA-identified exposed versus unexposed children. However, in children, higher CO levels were associated with decreased % predicted FEV_1_/FVC, *r* = −0.54, *p* = 0.010.

### 3.5. Reported Symptoms

**Figure 2:**
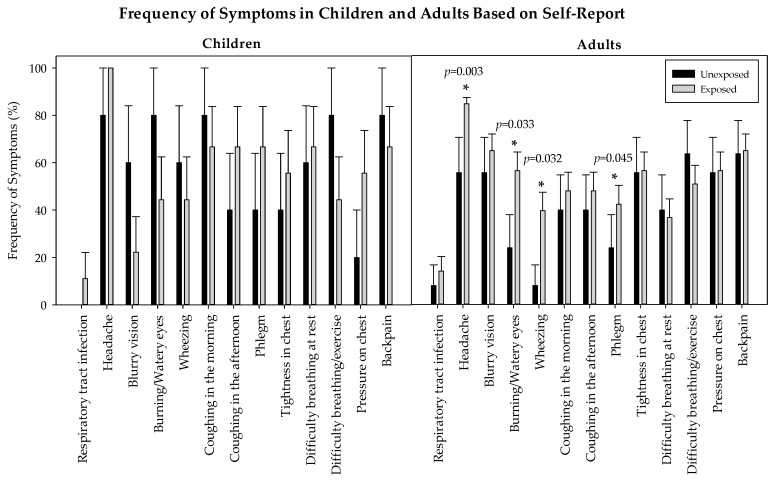
Reported respiratory and non-respiratory symptoms in children and adults. Unexposed adults did not have any reported exposure to HAP, and experienced significantly less headaches, burning and watering eyes, phlegm production and wheezing than adults who reported as being exposed to one or more HAP sources (*p* < 0.05). There were no significant differences between reported respiratory and non-respiratory symptoms in exposed adults and children (*p* > 0.05). Errors bars represent SD in sample population.

Adults who reported exposure to HAP were significantly more likely than adults not reporting exposure to experience headaches (96.8% versus 63.6%; *p* = 0.003); burning eyes (64.5% versus 27.3%; *p* = 0.033); wheezing (45.2% versus 9.1%, *p* = 0.032); and phlegm (48.4% versus 27.2%; *p* = 0.045) (Figure 2). Defined by CO cut points, exposed adults were significantly more likely than unexposed adults to experience blurry vision (89.5% versus 50%; *p* = 0.013). No differences in the frequency of respiratory symptoms were observed between exposed and unexposed children, *p* > 0.05, however exposure to smoking likely influences respiratory symptoms present in children that were not statistically significant (see limitations).

We conducted Fisher’s exact tests to determine whether the presence of respiratory symptoms differed between exposed adults who met PA guidelines versus those who did not. Adults who met PA requirements were significantly less likely than adults who did not meet these requirements to experience tightness (84.6% versus 42.9%) and pressure in the chest (85.7% versus 37.7%), *p* < 0.05. However, independent samples *t* tests indicated no differences between the two groups in objective measures of lung function (PFT’s), *p* > 0.05.

## 4. Discussion

### 4.1. Main Findings

The results of the present study do not support our primary hypothesis that increased HAP exposure in adults has deleterious effects on lung function. However, there was an association between reported HAP exposure and lower FEV_1_/FVC in children. The results support the hypothesis that elevated levels of HAP exposure are associated with increased reported respiratory and non-respiratory symptoms in adults, but not in children. Additionally, our second hypothesis that lifestyle behaviors may influence reported respiratory and non-respiratory symptoms was supported by the data in adults, but not in children.

### 4.2. Exposure to Household Air Pollution, Lung Function and Respiratory Health

Interestingly, although few differences were observed between exposed and unexposed children, children with higher CO tended to display decreased % predicted FEV_1_/FVC. For this lung function measure, a value below 70% is used to diagnose asthma and other obstructive diseases. Only one child had a FEV_1_/FVC below 70%, so although exposure may be associated with a significantly lower FEV_1_/FVC, this may not be clinically significant.

The association of HAP with PFT indices in the present work are consistent with previous research that has linked acute and chronic health effects to HAP exposure and CO [11,27,29]. Additionally, previous literature has reported children and adults with increased smoke exposure have greater non-respiratory symptoms (e.g., burning eyes and headache) [14] as well as greater prevalence of respiratory symptoms such as coughing and phlegm production [15]. Neither of these studies reported a reduction in PFT indices, even with high prevalence of reported symptoms.

Our results differ from previous research in that exposure did not negatively influence objective measures of lung health in adults. For example, Torres-Duque et al., (2008) present a body of previous research that has demonstrated mild to moderate reductions of FEV_1_/FVC, FEV_1_, PEF, and FEF_25%–75%_ associated with exposure to household biomass burning, adults in the present study did not differ on PFT measures as a function of exposure [30]. One likely contributor is that three adults in the exposed group had PFT values that were consistently greater than 150% of predicted. Additionally, the nature of walk-in clinics prohibited a familiarization day with the PFTs. Participants performed these tests according to ATS guidelines, and therefore we attempted to minimize variability by ensuring individuals could perform the tests consistently (within 10% of one another) and we took the best value for data analysis. Considering previous literature has determined that HAP exposure increases the risk ratios for a plethora of negative effects on respiratory health [31], it is important to interpret a field study with caution in extrapolating the findings to the general population. Effect size calculations revealed a stronger relationship between self-reported exposure and FEV_1_/FVC for children (*d* = −0.39, *p* = 0.05) than adults (*d* = −0.28, *p* = 0.05). Post-hoc power analyses indicated that 232 children and 534 adults would have been the necessary sample sizes to reveal significant decrements in FEV_1_/FVC with increased self-reported exposure.

### 4.3. Lifestyle as a Natural Antioxidant

One novel finding that we observed in the present work was that adults that were exposed to HAP and also met current physical activity requirements were significantly less likely to experience tightness and pressure in the chest than adults who were exposed and did not meet current PA requirements. It has been well established that physical activity may reduce the risk for asthma, airway hyperresponsiveness, and respiratory symptoms [22]. In fact, previous research suggests that the development of asthma is associated with decreased PA in both children [32] and adults [33]. Higher amounts of HAP have been reported to up-regulate oxidative [34,35] and inflammatory pathways [36]. However, lifestyle (FV consumption and meeting PA recommendations) may attenuate the increase in oxidative stress and inflammation when exposed to higher amounts of pollution [37,38,39]. While we did not directly measure the mechanisms through which FV and PA protect lung function when exposed to HAP, protective effects are likely to be a result of direct and indirect enzymatic mechanisms [40] that neutralize the harmful effects of free radicals [41]. These mechanisms may protect the lung from the free radicals that are produced when exposed to HAP. Future research is needed to clarify the extent to which PA behavior moderates the influence of HAP exposure on respiratory health in locations where there is both high HAP exposure as well as elevated outdoor air pollution.

### 4.4. Future Research Directions

Our data suggest that interventions to increase awareness and minimize HAP exposure among Belizeans may be needed. Anecdotally, many Belizeans were not aware about exposure sources, even when they had exhaled CO above the EPA cut point of 9 ppm. In addition to conducting interventions to reduce HAP, the low number of participants in the present study who met fruit and vegetable consumption guidelines (8.9%) suggests that dietary interventions may also be needed in this population. Bermudez et al., (2003) state that fruit and vegetable consumption is very low in Central American countries and is a major concern because it increases the total burden of disease [42]. Moreover, because fruit and vegetable consumption may lower inflammatory markers and provide antioxidants to combat oxidative stress [16] occurring due to HAP exposure, increasing FV consumption may mitigate effects of HAP in studies where the primary goal of decreasing HAP exposure is not achieved.

### 4.5. Limitations

Because of the nature of our study, we were not able to include a direct measure of the magnitude of exposure to HAP. Based on previous research suggesting that the harm caused by HAP’s may differ based on proximity to the HAP source, as well as the duration and intensity of exposure [3], direct measures would have been desirable. As noted above, however, breath CO output is an acceptable proxy for PM exposure, irrespective of its source [11], although not frequently used when direct measures are available. It would also have been desirable to determine the contributions from each exposure source (i.e., stoves, factory work, mosquito coils, tobacco); however, the primary aim of the field study was to determine differences in lung function and symptoms in adults and children classified as exposed compared to unexposed. Due to the nature of this field-based study, our sample size did not provide the power to detect differences between the contribution of each exposure source (mosquito coils, traditional wood-burning stoves, passive/active tobacco smoking) on respiratory symptoms and lung function.

Several families indicated that they had walked to the clinics, and therefore it is possible that outdoor air pollution contributed to the total exposure of these participants. We were not able to assess outdoor air pollution at the clinics. However, for this reason, we analyzed the data based on both EPA cut-points and self-reported measures of exposure in their household. Self-reported measures were included to allow assessment of whether symptoms and lung function differed based on which sources participants were exposed to, and not solely on their CO output. Considering CO output may change based on recent exposure, for example, when an individual was ill and could not attend work, CO may be elevated due to the individual having spent more hours in their home than normal. Also, the walk-in clinics could get very busy, and several participants left before completing all measures, which resulted in missing data. Regretfully, we do not have data on the individuals that left the clinic, however anecdotally the majority of the individuals that did not want to participate in the research either felt the line was too long to wait for pulmonary function testing, or they only attended the clinics to receive vitamins, supplementation and anti-inflammatories, which they received from the medical doctor. Due to the many individuals that did not agree to participate, the individuals that did complete testing may have been the most concerned about their health, possibly not representing the Belizean population. Still, hopefully this field study will shine some light specifically on Belize, which has not been previously studied using direct interventions and primarily included in the epidemiological literature. It is important to do further studies to determine whether lifestyle modification may improve health and quality of life in Belizeans.

Another important limitation is that this study included smokers, as smoking is associated with respiratory issues and increased exhaled CO [43]. In our sample, there were eight adult smokers and nine non-smokers exposed to secondhand smoke. An additional three children were exposed to secondhand smoke. In children, all children exposed to secondhand smoke reported coughing at night, phlegm production, tightness in the chest and having difficulty breathing during exercise, however no symptoms were statistically significant in children based on exposure. Surprisingly in adults, adults who smoked or lived with a smoker only reported more problems with vision and difficulty breathing during exercise, but didn’t differ on any other respiratory or non-respiratory measure. We have dichotomized groups by ((1) smokers/smokers in the house and (2) non-smokers) to show the effect size and *p*-value between the mean number of self-reported respiratory symptoms, non-respiratory symptoms, and also the mean FEV_1_/FVC in each group. Effect size calculations showed a small-moderate effect for smoking on FEV_1_/FVC (d = −0.21, *p* = 0.389), a moderate effect for smoking on respiratory symptoms (*d* = 0.32, *p* = 0.260) and a small effect for smoking on non-respiratory symptoms (*d* = 0.09, *p* = 0.731). Therefore, in this sample, it appears smoking did not have a large effect. However, if extrapolated to the entire Belizean population our results may differ. To report decrements in FEV_1_/FVC based on smoking status (either being a smoker or having a smoker in the house), 832 participants would be needed, 388 participants would be needed to show significant differences in respiratory symptoms, and 4530 would be needed to show significant increases in non-respiratory symptoms based on being a smoker or living in the house with a smoker. Finally, many children could not complete the PFTs properly without a familiarization trial and the consequent small sample may have prohibited our ability to detect significant relationships/differences in the children. While the sample size in this study may be too small to detect significant differences among several variables, these data collected from the walk-in clinics suggests there is a need to further investigate HAP exposure, pulmonary function and lifestyle in Belizeans adults and children.

## 5. Conclusions

Exposure to multiple CO sources contributes to a rise in respiratory symptoms among adults and children, though lifestyle variables may attenuate several negative consequences from HAP exposure. Our results also suggest that key points for intervention will be increasing Belizeans’ awareness about HAP sources, and providing opportunities to either (a) minimize exposure (e.g., not burn mosquito coils next to bed while sleeping); and/or (b) modify behavior (e.g., increase PA and FV consumption), such that the deleterious effects of HAP on lung health may be minimized. Because HAP accounts for a substantial portion of the global burden of disease in developing countries [4], future research accomplishing the above goals is likely to contribute substantially to improving public health in Belize and other developing countries.

## Figures and Tables

**Table 1:** Children and adults’ demographic data.

	Children (*n* = 23)	Adults (*n* = 67)
Variable	Mean ± SD	Mean ± SD
Age (years)	11.7 ± 3.1	44.2 ± 16.1
Sex (M/F)	6/17	19/48
Site		
Kontiki	4	8
Awe Village	3	12
Santiago	4	12
Basckstreet	4	14
Santa Elena	3	5
San Ignacio	3	8
Cayo	0	5
Santa Maria	2	3
Height (cm)	144.8 ± 17.5	158.8 ± 11.4
Weight (kg)	39.9 ± 12.3	76.1 ± 16.6
BMI (kg/m^2^)	18.0 ± 2.1	30.4 ± 6.1
Blood pressure (systolic/diastolic)	N/A	130/80 ± 20/12
Physical Activity guidelines		
Meets/Does not meet	9/14	31/36
Fruit and Vegetable Consumption		
Meets/Does not meet	1/22	5/62

Values are mean ± SD. BMI, body mass index. N/A, not applicable.

**Table 2:** Lung function data in self-reported unexposed versus exposed adults.

	Unexposed (*n* = 10)	% of Predicted	Exposed (*n* = 31)	% of Predicted	*p*-Value
	Mean SD	Mean SD	Mean SD	Mean SD	
FEV_1_ (L)	2.5 ± 0.6	91.0 ± 5.6	2.7 ± 0.9	100.1 ± 14.6	0.063
FVC (L)	2.9 ± 0.8	87.6 ± 6.5	3.3 ± 1.2	99.0 ± 19.7	0.066
FEV_1_/FVC (%)	87 ± 2.7	103.6 ± 6.3	82.0 ± 16.4	100.8 ± 12.5	0.510
FEF_25%–75%_ (L/s)	3.3 ± 0.6	100.8 ± 20.7	3.4 ± 1.4	103 ± 34.6	0.903
PEF (L/s)	5.1 ± 1.5	84.3 ± 22.9	5.3 ± 2.1	81.3 ± 19.7	0.693

Values are mean ± SD. There was no significant difference in lung function between unexposed and exposed adults (*p* > 0.05). *p*-values are calculated from the percent of predicted reference values.

**Table 3:** Lung function data in self-reported unexposed versus exposed children.

	Unexposed (*n* = 4)	% of Predicted	Exposed (*n* = 8)	% of Predicted	*p*-Value
	Mean SD	Mean SD	Mean SD	Mean SD	
FEV_1_ (L)	2.6 ± 0.6	89.8 ± 14.4	2.1 ± 0.6	87.8 ± 15.5	0.834
FVC (L)	2.8 ± 0.5	86.3 ± 10.3	2.3 ± 0.5	86.8 ± 14.8	0.953
FEV_1_/FVC (%)	91.9 ± 3.6	104.0 ± 3.7	91.0 ± 10.7	100.3 ± 12.8	0.595
FEF_25%–75%_ (L/s)	3.5 ± 0.7	105.3 ± 13.6	2.9 ± 1.1	93.8 ± 26.5	0.440
PEF (L/s)	4.8 ± 0.6	89.3 ± 16.4	4.1 ± 1.4	82.3 ± 18.3	0.534

Values are mean ± SD. *p*-values are included from the percent of predicted reference values.
